# Inference and forecast of H7N9 influenza in China, 2013 to 2015

**DOI:** 10.2807/1560-7917.ES.2017.22.7.30462

**Published:** 2017-02-16

**Authors:** Ruiyun Li, Yuqi Bai, Alex Heaney, Sasikiran Kandula, Jun Cai, Xuyi Zhao, Bing Xu, Jeffrey Shaman

**Affiliations:** 1 State Key Laboratory of Remote Sensing Science, College of Global Change and Earth System Science, Beijing Normal University, Beijing, China; 2 Department of Environmental Health Sciences, Mailman School of Public Health, Columbia University, New York, United States; 3 Ministry of Education Key Laboratory for Earth System Modeling, Center for Earth System Science, Tsinghua University, Beijing, China

**Keywords:** H7N9, avian influenza, data assimilation, forecasting

## Abstract

The recent emergence of A(H7N9) avian influenza poses a significant challenge to public health in China and around the world; however, understanding of the transmission dynamics and progression of influenza A(H7N9) infection in domestic poultry, as well as spillover transmission to humans, remains limited. Here, we develop a mathematical model–Bayesian inference system which combines a simple epidemic model and data assimilation method, and use it in conjunction with data on observed human influenza A(H7N9) cases from 19 February 2013 to 19 September 2015 to estimate key epidemiological parameters and to forecast infection in both poultry and humans. Our findings indicate a high outbreak attack rate of 33% among poultry but a low rate of chicken-to-human spillover transmission. In addition, we generated accurate forecasts of the peak timing and magnitude of human influenza A(H7N9) cases. This work demonstrates that transmission dynamics within an avian reservoir can be estimated and that real-time forecast of spillover avian influenza in humans is possible.

## Introduction

Wild birds, particularly *Anseriformes* and *Charadriformes*, are thought to be the principal natural reservoir of low pathogenic avian influenza (LPAI) viruses [1,2], as well as the source of influenza A viruses infecting all other animals [3]. Indeed, LPAI includes nearly all influenza subtypes, and wild bird migration can bring viruses to new areas and species [1,4]. The LPAI A(H7N9) virus was first identified in humans in China in early 2013 [5]. As at 15 October 2015, 678 confirmed human infections have been documented, with a case fatality rate of ca 40% [6]. The virus most probably originated in wild bird populations [7,8], was introduced into domestic ducks and chickens and has since become well established in poultry populations in south-eastern China [6]. Transmission to humans occurs primarily at live bird markets (LBMs), where direct contact between humans and infected poultry leads to spillover transmission [9].

Human influenza A(H7N9) infections have been well documented by the Chinese government and public health authorities. Outbreaks of human influenza A(H7N9) cases peak in winter months [10] and geographical diffusion from the eastern to the southern region of China has been observed [11]. As is true for most LPAI viruses, influenza A(H7N9) does not produce significant illness in domestic poultry, implying that poultry can be infected asymptomatically [6]. Consequently, poultry infections are likely to be under-reported even though LBMs are being closely and actively monitored [12,13]. This limited, partial observation of influenza A(H7N9) infection in poultry poses a challenge to the study and quantification of the transmission potential of H7N9 viruses in poultry populations, as well as spillover transmission from poultry to humans. However, owing to the transmission link between influenza A(H7N9) infection in poultry and human infection through LBMs [9], and because human influenza A(H7N9) cases have been well documented, these human cases serve as a sentinel proxy for infection rates among domestic poultry.

Mathematical approaches can be used to infer critical epidemiological processes and parameters. Traditional methods of epidemic curve fitting regard the increase in cumulative cases as an exponential with set doubling times [14]. This approach uses surveillance data during the early exponential growth period of an outbreak to provide retrospective estimates of *R*_0_ [15,16]. However, these estimates rely on specific assumptions, such as the initial susceptibility of the population and the infectious period. In contrast, a Bayesian approach [17] can provide continuous estimation of all system parameters without specific assumptions and is therefore more suitable for nonlinear epidemic modelling. In previous work, we used Bayesian inference methods to infer disease transmission dynamics, estimate critical epidemiological parameters, and generate forecasts of seasonal and pandemic human influenza (i.e. H1N1, H3N2, B) in both temperate [18-20] and subtropical regions [21].

**Figure 1:**
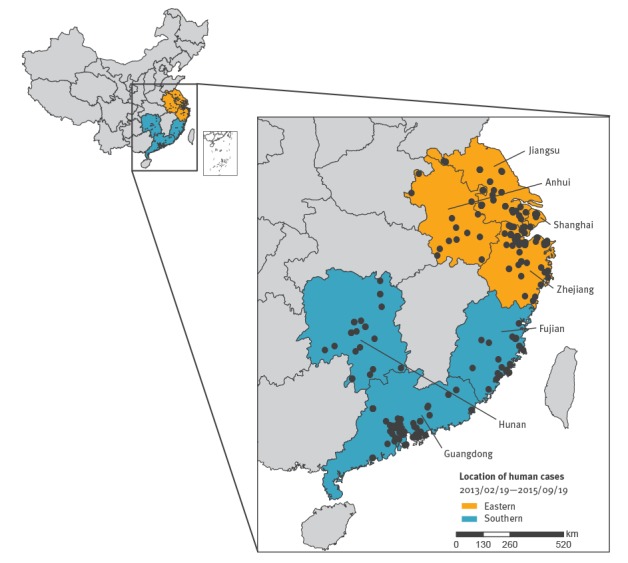
Spatial distribution of human influenza A(H7N9) cases and classification of study regions, China, 19 February 2013–19 September 2015 (n = 526)

Here we used human case data and a combined framework of mathematical model and Bayesian inference to simulate influenza A(H7N9) virus transmission among poultry and generate retrospective forecasts of influenza A(H7N9) incidence for both poultry and humans in the eastern and southern regions of China (Figure 1).

The human influenza A(H7N9) cases (black dots indicate case locations) in the study area are grouped into the eastern region (Jiangsu, Zhejiang, Shanghai and Anhui provinces: pink) and the southern region (Guangdong, Fujian and Hunan provinces: blue). These cases are shown in the whole map of China and the enlarged map of the study area. Also shown is the South Sea but with no human cases.

Specifically, human influenza A(H7N9) case data in the period from 2013 to 2015 were used in conjunction with a model-inference framework that combines a susceptible-infected-recovered (SIR) compartmental model of influenza A(H7N9) virus transmission among poultry and the ensemble adjustment Kalman filter (EAKF) to simulate influenza A(H7N9) virus transmission among poultry, estimate critical epidemiological parameters, and generate forecasts of influenza A(H7N9) infections for both humans and poultry. 

## Methods

### Data

From 19 February 2013 until 19 September 2015, a total of 526 human influenza A(H7N9) cases were extracted from official reports of the National Health and Family Planning Commission (NHFPC) in China. Associated record attributes included location, observation and reporting date, and descriptive information including age, sex and contact history.

These records were processed into biweekly counts during the 2012/13, 2013/14 and 2014/15 seasons and aggregated into two spatial regions, the southern region (Guangdong, Fujian and Hunan provinces) and the eastern region (Jiangsu, Zhejiang, Shanghai and Anhui provinces). This spatial grouping was based on the geographical location, common sources for poultry and virus spatial transmission patterns among the provinces. Specifically, provinces in the same region are geographically conjoined, and influenza A(H7N9) virus appeared to diffuse from the eastern region, where chicken farming and consumption occur locally, to the southern region where chickens are imported from northern China (e.g. Hebei and Shandong provinces).

### Description of the epidemical model

The epidemical model used for this study simulates the transmission of influenza A(H7N9) among poultry as well as spillover transmission from poultry to humans. The model is described by the following equations:

$$\frac{dS}{dt}=-\frac{\beta I_{c}S}{N}$$ (1)

$$\frac{dI_{c}}{dt}=\frac{\beta I_{c}S}{N}-\frac{I_{c}}{D}$$ (2)

$$I_{h}=\frac{I_{c}}{\gamma }$$ (3)

where *S* is the number of susceptible poultry, *I_c_* and *I_h_* are the number of infectious poultry and humans, respectively, *N* is chicken population size, *β* is the contact rate among poultry, *D* is the mean infectious period, and *γ* is the scaling factor linking the number of infected poultry with human infections. The basic reproductive rate, *R_0_*, is calculated from the infection rate and mean infectious period as *R_0_ = β D*, while the effective reproductive rate is also determined from susceptibility as *R_e_ = R_0_ S / N*.

This modelling framework was implemented with the assumption of homogenous mixing among chicken and human populations, indicating that spillover transmission from poultry to human was constant through time and that no transmission among humans occurred. In essence, we used human influenza A(H7N9) case data as a proxy for infection among poultry. We took this approach because infections among poultry are likely to be greatly under-reported and human influenza A(H7N9) incidence data are much more reliable.

### Description of the ensemble adjustment Kalman filter

The EAKF is a sequential Monte Carlo, or data assimilation, method that is used to iteratively update the model state variables and parameters with each new observation [22]. This update follows Bayes’ rule:

$$p(Z_{t}|y_{t},y_{t-1},\ldots )\propto p\left(y_{t}|Z_{t}\right)p(Z_{t}|y_{t-1},\ldots )$$ (4)

where *Z_t_* is the system state, including model variables and parameters *S*, *I_c_*, *I_h_*, *D*, *R_0_*, and *y_t_* is the observation at time *t*. Formula 4 shows that the updated (i.e. posterior) probability distribution is proportional to the product of the likelihood of the occurrence of new observations given the current system state and the prior probability distribution of the system state. The EAKF uses an assumption of normality for the likelihood and prior distribution. In doing so, only the first two statistical moments are needed to characterise the distributions on the right hand side of Formula 4.

The EAKF was selected for iterative Bayesian inference in this work because it was already being used for state space estimation in the geosciences (e.g. climate and weather simulation and prediction) and also in conjunction with influenza state space models to generate seasonal influenza forecasts [18,19,21].

All simulations of influenza A(H7N9) transmission and incidence among poultry and spillover transmission to humans with the model-inference system (i.e. the SIR dynamic model and EAKF inference) were run using a 300-member ensemble of simulations. These simulations were run simultaneously and linked through the EAKF. Before integration with the model equations, each simulation (i.e. ensemble member) was randomly assigned an initial combination of state variables and parameters from specified uniform distributions (see below). These comprised the initial conditions, or ‘initial prior’, for each simulation before integration. Each initialised ensemble member was then integrated through time using the equations of the model; as each simulation has a different initial array of state variables and parameters, the trajectory of each simulation differs. The ensemble was integrated until the time point of the first observation at which the run was halted and the EAKF algorithm and observation were used to update the ensemble mean and variance of the observed state variable (here incidence) according to Formula 4, as well as all the unobserved variables and parameters [19,22]. The conditions upon halting before the EAKF update are termed the ‘prior’; the conditions after the EAKF update are termed the ‘posterior’. The mean prior and posterior are averages across the ensemble; for example, the mean prior and posterior of susceptibility (*S*) is simply the ensemble average value of *S* before and after EAKF updating at a particular point in time.

The use of an ensemble of simulations provided an easy means of estimating credible intervals and uncertainties, both for parameter estimates and forecasts. Indeed, for the EAKF, the prior and posterior moments (i.e. mean and variance) can be calculated directly from the average prior and posterior estimates of all the 300 ensemble members.

The described cycle of integration and adjustment was repeated for each successive observation, i.e. after updating, the posterior was integrated through time until the next observation, at which point it became the prior. Then the EAKF and observation were used to generate a new posterior. Through this iterative updating process, the estimates of the state variables and parameters converge to a combination capable of simulating the outbreak as observed up to that point. The intention was that by optimising the model to simulate conditions as observed from the past to present, a better forecast of the future can be generated using that optimised ensemble of simulation.

### Initialisation and simulation with the SIR-EAKF framework

The state variable-parameter vector of the SIR-EAKF framework included optimisation of three variables (*S*, *I_c_* and *I_h_*) and two parameters (*D* and *R_0_*). At the beginning of each outbreak, we initialised each simulation (i.e. each ensemble member) using a random selection from uniform ranges of the parameters and variables (2 < *D* < 10 days, 0.01 < *R_0_* < 2.0, 0.5 < *S_0_* < 0.6, 0 < *I_c0_* < 250). These initial uniform ranges were based on prior modelling efforts simulating and forecasting human seasonal influenza [18,20]. In addition, as the transmission potential of influenza A(H7N9) virus among poultry is not well described, a broad initial prior range for *R*_0_ was used; however, note that the EAKF in the presence of observations can adjust the model parameters and variables to values outside these initial ranges. A Latin hypercube sampling approach was used to generate a near-random initial prior sample across this multidimensional distribution of parameter and variable values.

Multiplicative inflation was used to increase the ensemble variance of all model variables and parameters by 2% before EAKF adjustment. Inflation is commonly applied to ensemble Kalman filters in order to avoid ‘filter divergence’, the situation in which the variance across the ensemble of simulations has contracted so much that the EAKF updating algorithm effectively ignores the observations and model simulations diverge from the truth [22]. The 300-member ensemble simulations were repeated 10 times each season to account for stochastic effects due to the random selection of initial conditions. The average of the 10 repeated runs, each made up of a 300-member ensemble simulation, was used to derive mean posterior estimates of the model parameters.

## Parameter estimation

Several epidemiological parameters are critical for characterising the transmission potential of infectious diseases. The basic reproductive number *R*_0_, defined as the number of secondary infections an infectious host would produce in a completely susceptible population, signals the potential of an infectious agent to start an outbreak as well as the transmissibility of a virus in the absence of intervention. The effective reproductive number *R*_e_ quantifies the transmission force during the actual outbreak and can be used to monitor the impact of control strategies. An *R*_e_ > 1 indicates epidemic growth, while an *R*_e_ < 1 indicates that sustained transmission cannot persist and that an outbreak will subside.

**Figure 2:**
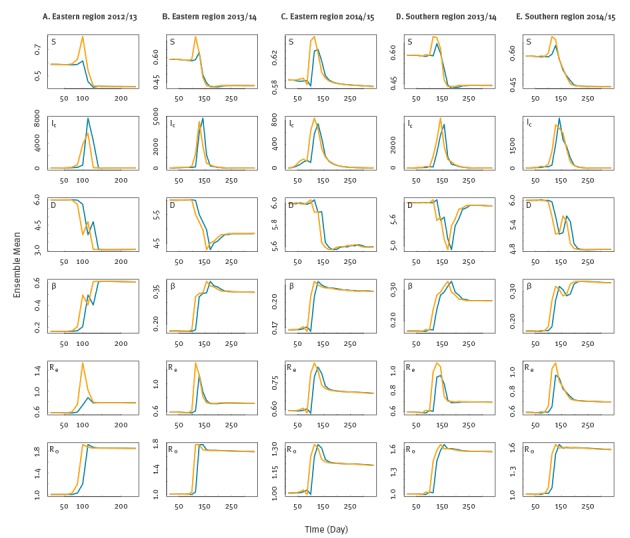
Parameter dynamics of H7N9 influenza across seasons for the eastern and southern region, China, 2012–2015

Epidemiological parameters, namely *β*, *D*, *R_0_* and *R_e_*, were estimated for each of three seasons and two regions from the start of the season to the last two-week period with a recorded case. In a given season, the posterior mean and interquartile range of *I_c_*, *I_h_*, β, *D*, R_0_ and R_e_ were estimated at the time of maximal epidemic forcing or the time point of highest transmission potential, i.e. the two-week period with the highest effective reproductive number. The level of initial susceptibility, however, was defined as and estimated for the two-week period with maximal susceptibility. We have previously presented parameter estimates at these key time points in studies of seasonal influenza [18,20]. The prior and posterior means during each outbreak for each variable and parameter were also recorded (Figure 2).

Time series of 300-member ensemble mean posterior (red) and prior (blue) for key epidemiological variables (susceptibility (*S*) and the number of infections in chicken (*I_c_*)), and for parameters (the infectious period (*D*), chicken-to-chicken contact rate (*β*) and the effective and basic reproductive rate (*R_e_* and *R_0_*)), during different seasons and regions. In each season, variables and parameters were adjusted from the beginning of the season to the two-week period with the last recorded case. Both the prior and posterior mean estimates are shown; EAKF adjustment at a given time is the difference of the mean posterior minus the mean prior.

Parameter estimate changes during the entirety of an outbreak were used to inspect filter adjustment. Such parameter changes over time may reflect changes in the estimation or actual changes to the parameter values. For the former, the observations contain noise and the estimation of the parameters by the EAKF is neither perfect nor instantaneous; consequently, the parameter estimates move through time. For the latter, actual shifts in parameter value can occur, e.g. representing changing contact rates and control measures, as the pathogen moves through different subpopulations and/or geographical areas.

### Sensitivity analysis

The parameter estimates were inferred using a scaling *γ*, representing a rate of spillover transmission from chicken to human, equal to 300. This value was selected following tests with *γ* ranging from 100 to 1,000 in increments of 100. For each value of *γ*, mean human case forecast error was used to calculate total outbreak root mean squared error (RMSE) and correlation, as well as attack rate error, peak weak error and peak magnitude error between observations and the predicted estimates. A ranking approach was used to identify the scaling with the lowest error. Specifically, for each metric (RMSE, correlation, attack rate error, peak weak error and peak magnitude error), the scaling levels were ranked. The scaling with the highest overall rank, i.e. *γ* = 300, was selected and used in all simulations and forecasts presented here.

### Retrospective forecasts

Retrospective forecasts were run for the seasons 2012/13, 2013/14 and 2014/15 for the eastern region and for the last two seasons for the southern region. The model-inference system was again implemented using 300-member ensembles and reinitialised with randomly selected variable and parameter combinations at the beginning of each season. All simulations and forecasts were repeated 10 times for each outbreak and were initialised with a random selection of parameter and variable values, as described above. Forecasts were generated beginning with the two-week period of the first recorded case and repeated every 2 weeks following the generation of a new posterior. Specifically, for the eastern region, separate ensemble forecasts were run from the 4th to 9th, 2nd to 17th and 3rd to 9th two-week period for the 2012/13, 2013/14 and 2014/15 seasons, respectively; for the southern region, forecasts were generated from the 3rd to 19th and 5th to 12th two-week period for the last two seasons.

To evaluate the accuracy of our SIR-EAKF system, we determined two measurements: the peak week and peak magnitude, or the percentage of ensemble mean trajectories predicting human influenza A(H7N9) case peak timing within ± 1 week of the observed peak week, and peak magnitude within ± 25% of the observed peak magnitude. These two indices were then plotted as a function of the relative forecast week, i.e. the week of forecast generation minus either the observed or predicted peak week, to show the relationship between predictive skill and lead time.

The combined SIR-EAKF system was coded in R. These codes are available from the corresponding author upon request.

## Results

**Figure 3:**
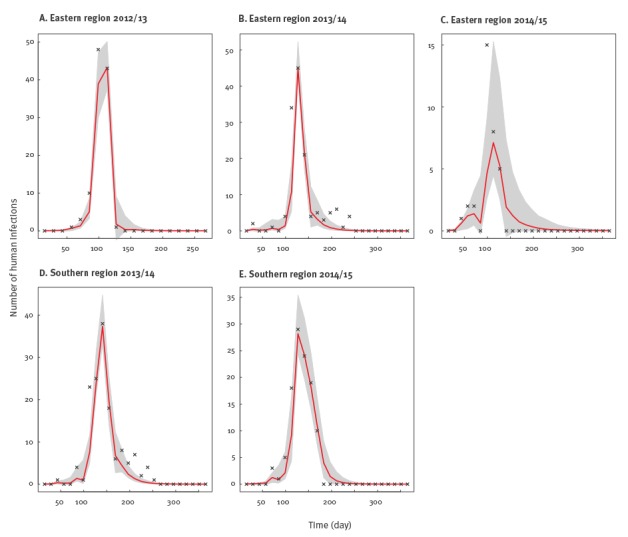
SIR-EAKF simulations of human H7N9 influenza across seasons and regions, China, 2012–2015

The mean posterior estimates of human influenza A(H7N9) incidence produced by the model-inference system matched the observed influenza A(H7N9) human case counts well (Figure 3).

300-member ensembles are simulated and repeated 10 times for each season. Initial conditions were varied with each repetition. The ensemble mean prior (blue) and posterior (red) estimates and the corresponding 5th and 95th percentiles of the ensemble posterior estimates of human infections were averaged over all 10 runs and compared with observed case numbers (black). The area between the 5th and 95th percentiles of prior and posterior estimates is shaded blue and grey, respectively.

These simulations captured the timing and magnitude of the epidemic. Mean posterior estimates of *R_0_* ranged from 1.327 to 1.941 (Table 1) with the highest and lowest estimates occurring in seasons with the largest and smallest numbers of human cases, i.e. the 2012/13 and 2014/15 seasons in the eastern region, respectively. The mean infectious period *D* was estimated at 5 to 6 days for outbreaks during the seasons 2013/14 and 2014/15. For the first human influenza A(H7N9) outbreak in 2012/13 in the eastern region, the estimate for *D* was much lower (mean: 3.95 interquartile range (IQR): 3.76–4.13) and the estimate of *β*, the contact rate among poultry, was higher (mean: 0.49/day; IQR: 0.47–0.51/day).

**Table 1:** Estimates of key epidemiological parameters and variables for H7N9 influenza, China, 2012–2015

Region	Season	*R_e_* maximum (IQR)	*R_0_* at maximal *R_e_* (IQR)	*D* at maximal *R_e_* (IQR)	*β* at maximal *R_e_* (IQR)	* S* maximum % (IQR)
Eastern (Jiangsu, Zhejiang, Shanghai, Anhui)	2012/13	1.56 (1.53–1.59)	1.94 (1.92–1.96)	3.95 (3.76–4.13)	0.49 (0.47–0.51)	80.72 (79.34–82.47)
	2013/14	1.34 (1.30–1.38)	1.81 (1.79–1.83)	5.69 (5.37–6.05)	0.32 (0.30–0.33)	73.98 (72.41–75.51)
	2014/15	0.86 (0.84–0.87)	1.32 (1.31–1.33)	5.94 (5.90–6.02)	0.22 (0.21–0.23)	64.86 (64.19–65.55)
Southern (Guangdong, Fujian, Hunan)	2013/14	1.08 (1.04–1.09)	1.59 (1.56–1.63)	5.60 (5.41–5.69)	0.28 (0.27–0.29)	69.45 (68.80–70.37)
	2014/15	1.06 (1.05–1.07)	1.62 (1.61–1.64)	5.29 (5.18–5.43)	0.31 (0.30–0.32)	68.94 (67.01–70.54)

IQR: interquartile range.

The posterior means and IQR of the number of chicken infections (*I_c_*), chicken-to-chicken contact rate (*β*), the infectious period (*D*) and the basic reproductive rate (*R_0_*) were estimated at maximal epidemic forcing (maximal *R_e_*). The level of initial susceptibility (*S*) was defined and estimated in the two-week period with maximal susceptibility.

The susceptibility of the chicken population was high in earlier outbreaks and dropped to around 65% in more recent outbreaks. For the effective transmission number *R_e_*, which quantifies the transmission force during the outbreak, the mean posterior estimates were greater than 1 during four of the five outbreaks analysed here, indicating a clear transmission potential among LBM poultry. The *R_e_* estimate was highest during the initial outbreak in 2012/13 when the two associated parameters, *R_0_* and susceptibility, were also highest. The scaling factor *γ*, selected by the rank correlation approach (see Methods) mapped the observed human cases to simulated poultry infections and indicated that the daily poultry-to-human spillover transmission rate was low, around 3.3 × 10^-3^ per infected LBM chicken.

Estimates of all parameters remained stable during the seasons 2013/14 and 2014/15 in the southern region where outbreaks were of similar severity in both epidemic waves. However, there was an apparent decrease in *R_0,_ R_e,_ β* and susceptibility from the first to the third outbreak in the eastern region, which was in accordance with the change of outbreak severity in this region.

**Figure 4:**
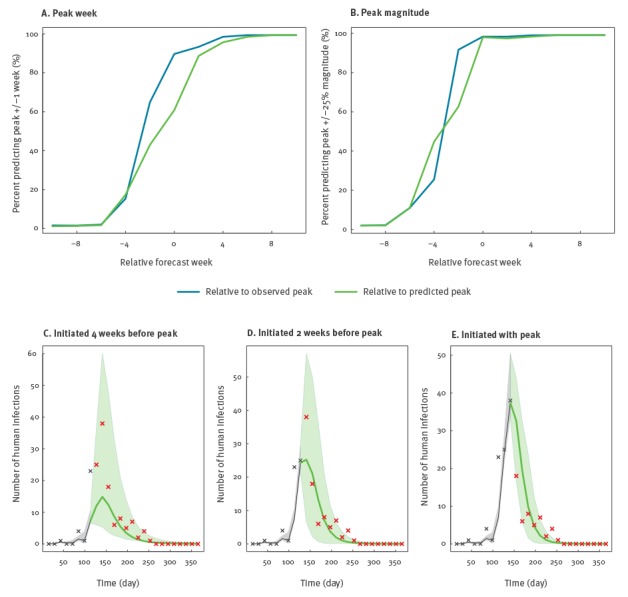
Forecast accuracy for all seasons and example forecasts of H7N9 influenza in the southern region, China, 2013/14 season

The accuracy of the forecast for peak timing and magnitude increased as the week of forecast initiation got closer to the observed and predicted peak (Figure 4).

Top panels: Accuracy was measured as the percentage of ensembles predicting (A) the week with the most human influenza A(H7N9) cases within one week of the observed peak week and (B) the peak magnitude of human influenza A(H7N9) cases within 25% of the observed peak magnitude. It is presented as a function of the forecast week relative to observed (blue) and predicted (green) peak timing.

Bottom panels: Forecasts initiated (C) 4 weeks, (D) 2 weeks and (E) 0 weeks ahead of the observed peak week for the southern region during the 2013/14 season. The SIR model was recursively optimised up to the week of forecast initiation using observations (black x) and the EAKF; the red x are future observations which were not used in the model optimisation. The black and green lines are the mean trajectory of the ensemble and areas shaded grey and green are the 5th and 95th percentiles of the ensemble posterior for simulation and forecast periods, respectively.

Specifically, the percentage of forecasts predicting the peak week within ± 1 week increased sharply from 6 weeks ahead of the observed peak week and reached 90% when a forecast was generated at the observed peak. For peak magnitude, the percentage of forecasts predicting the peak magnitude within ± 25% of the observed magnitude increased from 8 weeks before the observed peak, and almost all forecasts were accurate when predicting at the observed peak. However, as knowledge of the observed peak was unavailable for real-time forecasting, we also present overall accuracy as a function of predicted lead time. Here, the accuracy was 43% and 63% at 2 weeks lead time and 61% and 98% at 0 weeks lead time for peak timing and magnitude, respectively (Table 2). Example forecasts are also presented in Figure 4.

**Table 2:** Forecast accuracy for H7N9 influenza in all seasons, China, 2013–2015

	Relative forecast lead time (weeks)										
	−10	−8	−6	−4	−2	0	2	4	6	8	10
**Proportion predicting peak ± 1 week (%)**											
Relative to observed peak	1.59	1.50	2.00	15.33	64.84	89.67	93.33	98.50	99.34	99.34	99.34
Relative to predicted peak	1.17	1.33	1.67	17.33	42.92	60.83	88.67	95.67	98.50	99.34	99.34
**Proportion predicting peak ± 25% magnitude (%)**											
Relative to observed peak	2.00	2.17	11.00	25.50	91.67	98.33	98.33	99.00	99.08	99.08	99.08
Relative to predicted peak	2.00	2.00	11.00	44.75	62.67	98.00	97.42	98.33	99.08	99.08	99.08

Accuracy was measured as the percentage of ensembles predicting the week with the most human cases of influenza A(H7N9) within ± 1 week of the observed peak week and the peak magnitude of human H7N9 influenza cases within ± 25% of observed peak magnitude. The values are the same as those in Figure 4 and presented as a function of the forecast lead time from 10 weeks before to 10 weeks after the observed and predicted peak timing.

## Discussion

Our findings indicate that data assimilation methods and a simple epidemic model can be combined to infer the transmission dynamics of H7N9 influenza in both chicken and human populations using only human infection data. Moreover, the model-inference system can produce accurate predictions of the peak timing and magnitude of human infections.

The estimated potential of chicken-to-human spillover transmission was low, even with the high transmission rate among poultry. Specifically, estimates of *R_0_* were greater than 1 and the mean contact rate among poultry was 0.326 across all seasons and regions, whereas the daily chicken-to-human infection rate reflected by the linkage parameter *γ* indicated that the mean number of human infections per infectious chicken was 3.3 × 10^-3^. Our estimates of *R_0,_* among poultry were similar to those of past pandemic influenza viruses in humans (e.g. 1.2–2.3 for influenza A(H1N1)pdm09) [23], which implies that influenza A(H7N9) has the potential to cause pandemics in chicken populations. This result is similar to earlier findings [24]; however, our estimates for three other parameters, the mean infectious period, the basic reproductive rate and the chicken-to-human infection rate, were smaller, which may be due to the finer spatial and longer temporal scales used in this study, as well as the difference in modelling approach. Specifically, our study used a dynamic model, Bayesian inference framework and regional bi-weekly counts of human infections, covering three epidemic waves. Our findings thus represent more detailed, localised and long-term patterns of transmission dynamic than earlier work using least-square methods in conjunction with daily human infection data at the beginning of the outbreak at a national scale [24].

The dynamic patterns of influenza A(H7N9) differed in the two regions studied here, although with the limited number of outbreaks available for validation, these differences must be interpreted with caution. The transmission potential among chicken flocks and initial susceptibility decreased across three seasons in the eastern region, but remained stable in the southern region. These differences were dynamically consistent with observed outbreak severity in both regions and may have been caused by a difference in control methods implemented by the government. In the eastern region, approaches such as closing of LBMs [25,26] and halting live poultry trade were implemented during the early stages of the outbreaks. This probably reduced chicken-to-human exposure and chicken-to-chicken mixing and consequently may have attenuated the severity of the outbreak. On the other hand, for southern provinces such as Guangdong (where LBM closure was implemented later, in the second half of February 2014), co-circulation of a diverse array of avian influenza subtypes as well as multiple strains of H7N9 and H9N2 influenza viruses has been documented. This abundance of viruses creates an environment primed for influenza reassortment, resulting in diversified and more adaptive genotypes and a higher risk of infections in both poultry and humans [27,28] and may therefore keep susceptibility high and stable across seasons.

The mean estimate of *D*, the mean infectious period, for the 2012/13 outbreak in the eastern provinces was lower (3.95) than for the later outbreaks, which ranged from 5.29 to 5.94. Given the limited number of total outbreaks investigated, the exact causes for this difference are difficult to pinpoint; however, factors could include actual changes to the virus between the first and later outbreaks, errors in the observed number of cases or errors in the estimation process. That the 2013/14 and 2014/15 outbreaks yielded consistent estimates, including similar values for *D* and *β*, and decreasing maximal *S* over time suggests that these findings are credible.

Our inference and forecasting framework was implemented with a simple SIR model and the assumption of homogeneous mixing among human and chicken populations, i.e. a constant chicken-to-human transmission rate. Our model only simulated chicken-to-chicken and chicken-to-human transmission (Formulas 1–3) and did not consider environmental transmission. Given the limited data on infection and transmission among poultry, inferred distinctions of alternate transmission modes, i.e. chicken-to-chicken vs environment-to-chicken, are likely to be poorly constrained. Further, prior attempts to simulate these different pathways suggest that the rates of chicken-to-environment shedding are low [29]. Loss of immunity was not modelled either, as birds are either slaughtered or, when infection is suspected, culled, as required by the Chinese government [30].

Despite these shortcomings, the combined model-inference system matched the observations well, and provided sensible estimates of key epidemiological parameters, including rates of chicken-to-human spillover infection. The analyses revealed the transmission potential of H7N9 influenza among poultry, the stability and changes of that transmission potential over time, and that real-time forecasting of influenza A(H7N9) incidence in both human and poultry is possible. In the future, such methods could be applied in real time to newly emerged avian influenza subtypes.
